# Allergenicity Alleviation of Bee Pollen by Enzymatic Hydrolysis: Regulation in Mice Allergic Mediators, Metabolism, and Gut Microbiota

**DOI:** 10.3390/foods11213454

**Published:** 2022-10-31

**Authors:** Yuxiao Tao, Enning Zhou, Fukai Li, Lifeng Meng, Qiangqiang Li, Liming Wu

**Affiliations:** 1 Institute of Apicultural Research, Chinese Academy of Agricultural Sciences (CAAS), Beijing 100093, China; 2 Key Laboratory of Agro-Product Quality and Safety, Institute of Quality Standards and Testing Technology for Agro-Products, Chinese Academy of Agricultural Sciences (CAAS), Beijing 100081, China

**Keywords:** bee pollen, enzyme-treatment, allergenicity alleviation, metabolism, gut microbiota

## Abstract

Bee pollen as a nutrient-rich functional food has been considered for use as an adjuvant for chronic disease therapy. However, bee pollen can trigger food-borne allergies, causing a great concern to food safety. Our previous study demonstrated that the combined use of cellulase, pectinase and papain can hydrolyze allergens into peptides and amino acids, resulting in reduced allergenicity of bee pollen based on in vitro assays. Herein, we aimed to further explore the mechanisms behind allergenicity alleviation of enzyme-treated bee pollen through a BALB/c mouse model. Results showed that the enzyme-treated bee pollen could mitigate mice scratching frequency, ameliorate histopathological injury, decrease serum IgE level, and regulate bioamine production. Moreover, enzyme-treated bee pollen can modulate metabolic pathways and gut microbiota composition in mice, further supporting the alleviatory allergenicity of enzyme-treated bee pollen. The findings could provide a foundation for further development and utilization of hypoallergenic bee pollen products.

## 1. Introduction

Bee pollen as a natural food source is regarded as an excellent nutritional supplement for human consumption. It contains a variety of nutrients including proteins, carbohydrates, lipids, polyphenols, and many other nutrients [1]. Bee pollen also displays a variety of beneficial health properties, such as antioxidant, antibacterial, hepatoprotective, and cardioprotective activities [2,3]. By 2024, the global bee pollen market is expected to reach a value of USD 720 million according to Marketwatch.com. Global bee pollen consumption shows an upward trend with increasing numbers of consumers regarding bee pollen as a nutritional supplement. However, bee pollen consumption can cause a number of clinical allergic symptoms in certain individuals with allergies [4,5]. In addition, bee pollen can induce cross-allergic reactions when consumed with other foods [6,7,8]. The potential allergenicity of bee pollen has become one of the key issues limiting the development and utilization of bee pollen. Developing an efficient approach for reducing the allergenicity of bee pollen is thus necessary for expanding its further utilization.

Enzyme treatment is a critical food processing technique that can increase the nutritional value and reduce the allergenicity of foods. Furthermore, this technique is low cost and highly efficient [9]. Currently, α-chymotrypsin, trypsin and flavourzyme are widely used to reduce the allergenicity of certain allergenic foods, such as peanuts [10,11], soybean [11,12], and wheat [13]. Enzyme treatment has also been applied to break down the pollen wall and release nutrients contained within [14]. In our previous study, cellulase, pectinase and papain were combined and used for allergen degradation into small peptides and amino acids, resulting in decreased bee pollen allergenicity based on in vitro assays [15]. In this study, we aim to further clarify the mechanisms of allergenicity alleviation of enzyme-treated bee pollen on the changes in serum allergic mediators, metabolic pathways, and gut microbiota composition.

Immunoglobulin (Ig) E-mediated type I hypersensitivity accounts for the vast majority of food allergies [16]. As a high-IgE response strain, the BALB/c mouse is suitable for IgE-mediated food allergy research, such as egg- [17,18], milk- [19], fish- [20] and peanut- induced [21,22] hypersensitive reactions. Recently, BALB/c mice were adopted to study the pathogenesis of allergy syndrome caused by oral pollen [23], providing the basis for the selection of an in vivo model in our study. Additionally, food allergies can activate the release of cytokines such as interleukin(IL)-4, IL-5, IL-13, and other mediators that can induce the production of IgE antibody in B cells [16]. Food allergies can also cause changes in host metabolism and gut microbiota. Some studies have applied metabolomics to investigate IgE-mediated food allergies [24,25,26]. A close association between food allergies and the dysbiosis of gut microbiota was also proposed in numerous studies [27,28,29]. The immune system can be influenced by host metabolic disorders and gut microbiota dysbiosis, although the related mechanism remains unclear [30].

To further clarify the mechanisms behind allergenicity alleviation of enzyme-treated bee pollen, the BALB/c mouse model was used to investigate the regulatory effects of enzyme-treated bee pollen on the production of serum allergic mediators, changes in metabolic pathways, and gut microbiota composition. Bee pollen samples were treated with a combination of cellulase, pectinase and papain, and mice were fed enzyme-treated and non-enzyme-treated bee pollen, respectively. The scratching behavior and histopathological injury were evaluated to identify the allergic state in mice. Subsequently, the mice serum was collected for allergic mediator and metabolite assays. Mice fecal DNA was extracted for gut microbiota composition analysis. Our findings might provide a basis for further development and utilization of hypoallergenic bee pollen products.

## 2. Materials and Methods

### 2.1. Reagents and Apparatus

Imject^TM^ Alum Adjuvant (No. 77161) was obtained from Thermo Scientific Inc. (Pittsburgh, PA, USA). Ovalbumin (OVA) was obtained from Sigma-Aldrich Inc. (Saint Louis, MO, USA). BCA protein assay kit was obtained from Beyotime Biotechnology Co., Ltd. (Shanghai, China). Goat anti-mouse IgE antibody and HRP-labeled donkey anti-goat IgG antibody were obtained from Abcam Inc. (Cambridge, UK). DAB peroxidase substrate kit was purchased from Solarbio Co., Ltd. (China). The HPLC-grade acetonitrile, methanol, ammonium formate and formic acid were obtained from Fisher Scientific (Pittsburgh, PA, USA). Ultrapure water was collected from Millipore Milli-Q system (Bedford, MA, USA). Cellulase (400 U/mg), pectinase (500 U/mg) and papain (800 U/mg) were obtained from Yuanye Bio-Technology Co., Ltd. (Shanghai, China). Ultrafiltration centrifugal tube (15 mL, 10 kDa) was purchased from Millipore Inc. (Bedford, MA, USA). Other reagents were purchased from Sigma-Aldrich Inc. (Saint Louis, MO, USA).

### 2.2. Enzymatic Treatment of Bee Pollen

Bee pollen samples (composed of more than 92% *Brassica campestris* pollen according to palynological counting) were collected from the beekeeping base of the Institute of Apicultural Research (IAR), Chinese Academy of Agricultural Sciences (CAAS). The sample was lyophilized after grinding into powder, and then sterilized by irradiation at 7 kGy. Cellulase, pectinase and papain were used for enzymatically treating bee pollen as described in our previous study [15]. In brief, 10 mL of Millipore water and 5 g of bee pollen powder were combined and vortexed for 5 min. For two kinds of enzyme-treated bee pollen (2E-BP) groups, 3000 U cellulase and 3000 U pectinase were added into the sample. For three kinds of enzyme-treated bee pollen (3E-BP) groups, 3000 U cellulase, 3000 U pectinase and 3000 U papain were added into the sample. All samples were adjusted to pH 4.0 with vitamin C solution, and then incubated at 45 °C for 24 h. A vacuum freeze dryer was used for sample lyophilization, and samples were stored at −80 °C for further study.

### 2.3. Protein Sample Preparation

Bee pollen protein was extracted using water and 5% NaCl solution under ultrasonication for 45 min, respectively. The mixture was centrifuged at 3, 500 g for 15 min. The supernatant was then filtered with a nylon membrane (22 μm). The filtered solution was subsequently ultrafiltered using a Millipore ultrafiltration tube (15 mL, 10 kDa) at 3500 g for 30 min. The protein concentration of the ultrafiltration retentate was detected using a BCA protein assay kit.

### 2.4. Animal Experiment

**Figure 1:**
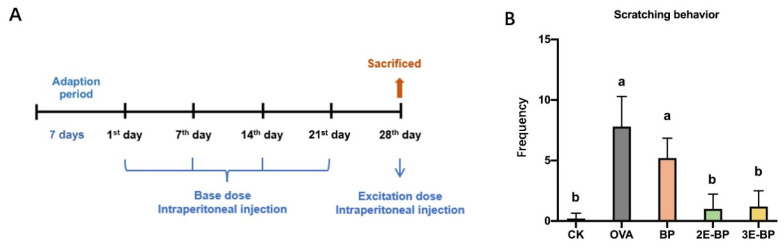
BALB/c mice administration schedule. (**A**) Scratching frequency of mice in different treatment groups. (**B**) Different letters indicate a significant difference among different groups (*p* < 0.05).

The animal experiments were conducted in Beijing Animal Experimental Center. The Animal Ethics Committee of IAR, CAAS (Beijing, China) provided approval for the animal experimentation. The registration number is CAAS-IAR-ER0046. Female BALB/c mice (6-week-old, 16–20 g) were kept in cages, housed in a 12-h light–dark cycle at 20–24 °C and a relative humidity of 50 ± 5%, and supplied with filtered pathogen-free air, standard AIN-93 laboratory diet and sterile water. After acclimation for one week, all mice were randomly divided into five groups (*n* = seven per group), and named as CK, OVA, BP, 2E-BP and 3E-BP. The mice administration procedure is shown in Figure 1A. Briefly, mice in all groups were given a standard diet and water. For the CK group, mice were intraperitoneally injected with a saline solution every seven days for 28 days; but for the OVA group (as a positive control), mice were injected intraperitoneally with 0.2 mL of 0.1 mg/mL OVA solution (containing 1% Alum Adjuvant) every seven days for 21 days, and finally injected with 0.2 mL of 0.5 mg/mL OVA solution (containing 1% Alum Adjuvant) on the 28th day; for BP, 2E-BP and 3E-BP groups, mice were injected intraperitoneally with 0.2 mL of 0.5 mg/mL BP, 2E-BP and 3E-BP solution (containing 1% Alum Adjuvant) every seven days for 21 days, respectively, and finally injected with 0.2 mL of 2.5 mg/mL BP, 2E-BP and 3E-BP solution (containing 1% Alum Adjuvant) on the 28th day, respectively. The frequency of scratching behavior was observed and recorded 15 min after final injection. Mice were sacrificed after intraperitoneal injection with an excitation dose of samples for 30 min on the 28th day. The blood was extracted from eyes, and then centrifuged at 3000 rpm for 10 min for serum collection.

### 2.5. Histopathological Testing

The formalin-fixed spleen tissue was embedded in paraffin. Next, the spleen tissue was sliced into sections and stained with toluidine blue (TB) and GIMSA separately. The sections were observed using a Nikon Eclipse Ci microscope (Tokyo, Japan).

### 2.6. Detection of Allergy Indexes in Mice Serum

#### 2.6.1. Detection of IgE Antibody in Mice Serum

The protein concentration in mice serum was detected by BCA protein assay kit. The serum (containing 40 μg protein) was added onto a nitrocellulose membrane (4 cm × 4 cm). After being dried, the membrane was incubated with 5% (*w*/*v*) BSA solution at 25 ± 2 °C for 1 h, and then incubated in 5% BSA solution containing 1:2000 diluted goat anti-mouse IgE antibody for 1 h. After washing the membrane with TBS-T solution, it was then incubated in 5% BSA solution containing 1:1500 diluted HRP-labeled donkey anti-goat IgG antibody for 1 h. After washing the membrane with TBS-T solution, the spots on the membrane were developed using a DAB color developing kit. Finally, the dried and developed membrane was imaged using an HP scanner, and the relatively quantitative analysis of dot intensity on the membrane was processed using Image J (Version 1.53).

#### 2.6.2. Bioamine Detection via UPLC-QQQ-MS/MS

To detect the changes of bioamines in mice serum, 50 μL serum was mixed with 200 μL methanol, and then centrifuged at 13,000 g for 15 min. The supernatant was filtered with a nylon membrane (22 μm). Samples were separated using an Agilent 1290 Infinity II series UPLC system equipped with an Agilent Zobax Eclipse C18 Rapid Resolution HD column (2.1 mm × 100 mm, 1.8 μm). Mobile phases A and B were water (containing 2 mM ammonium formate and 0.1% *v*/*v* formic acid) and methanol, respectively. The gradient was set as follows: 1 min, 2% B; 4 min, 15% B; 4.5 min, 98% B; 6 min, 98% B; 6.1 min, 2% B; post time 3 min, 5% B. An Agilent 6470 ESI-QQQ system was adopted for MS acquisition. Mass spectrometry parameters were set as follows: 250 °C, gas temperature; 7 L/min, drying gas flow rate; 30 psi, nebulizer pressure; 325 °C, sheath gas temperature; 11 L/min sheath gas flow rate. The precursor ion, product ion, collision energy and fragmentor are listed in Table S1 in Supplementary Materials.

#### 2.6.3. Metabolomics Analysis via UPLC-QTOF-MS/MS

To determine the changes of metabolites in mice serum, 50 μL serum was mixed with 200 μL methanol, and centrifuged at 13,000 g for 15 min. The supernatant was extracted and filtered with a nylon membrane (22 μm). Sample separation was accomplished using a UPLC system of Agilent 1290 Infinity II series. The UPLC system was equipped with an Agilent Eclipse Plus C18 Rapid Resolution HD column (2.1 mm × 100 mm, 1.8 m). The mobile phases and gradient were set as described [31]. For MS acquisition, an Agilent 6545 ESI-Q-TOF mass spectrometer was employed. The mass spectrum parameters were the same as our previous publication [31]. Reference ions 112.985587 and 1033.988109 were used for real-time calibration during acquisition in negative ionization mode. Metabolites were identified with the METLIN Database (DB). Metabolites with DB scores above 80 and mass error lower than 5 ppm (0.0005%) were screened as biomarkers for statistical analysis. Metabolic pathway analysis was conducted using MetaboAnalyst 4.0 and KEGG online platform.

### 2.7. Microbiota Analysis

The fecal DNA of mice was extracted using a commercial E.Z.N.A.^®^ DNA kit from Omega Bio-Tek Inc. (Norcross, GA, USA). DNA concentration and purity were determined via NanoDrop2000 from Thermo Fisher Scientific Inc. (Pittsburgh, PA, USA). The 1% agarose gel electrophoresis was used for DNA quality assay. A thermocycler PCR system (ABI GeneAmp^®^ 9700, Thermo Fisher Scientific, Waltham, MA, USA) was adopted for the amplification of 16 S rRNA gene in the hypervariable region of bacterial V3-V4, conducting with the universal primers 338F (5′-ACTCCTACGGGA GGCAGCAG-3′) and 806R (5′-GGACTACHVGGGTWTCTAAT-3′). An AxyPrep DNA gel extraction kit (Axygen Inc., Corning, NY, USA) was applied to purify the amplified products, and a Quantus^TM^ fluorometer (Promega Inc., Madison, WI, USA) was used for the quantification of the purified products. An Illumina MiSeq System (Illumina Inc., San Diego, CA, USA) was adopted for the paired-end sequencing of amplicons. The raw Illumina data was quality-filtered and merged using FASTQ version 0.20.0 software and FLASH version 1.2.7 software, respectively. Sequences with more than 97% similarity were clustered into the same amplicon sequence variants (ASVs) by DADA2 plugin of Qiime2 version 2020.2 software. The ASVs taxonomic assignments were performed using the naïve Bayes consensus taxonomy classifier based on SILVA 16S rRNA database (v 138). The analysis of 16S rRNA microbiome sequencing data was conducted by bioinformatic tools on Majorbio i-Sanger cloud platform (http://en.majorbio.com). The alpha-diversity and beta-diversity analysis, Kruskal–Wallis H test, and linear discriminant analysis effect size (LEfSe) analysis were performed based on the Majorbio i-Sanger cloud platform.

### 2.8. Data Analysis

The variance t-test and ANOVA analysis were performed using SPSS version 21.0 software at a 95% confidence level. The Multi-experiment viewer (MEV) version 4.9 software was applied to heat-map analysis.

## 3. Results and Discussion

### 3.1. Enzyme-Treated Bee Pollen Alleviates Mice Scratching Behavior

The itch-associated response is a typical allergic reaction and can be used as an indicator for the evaluation of anaphylaxis levels [32,33]. To reflect the level of anaphylaxis, scratching frequency of mice was recorded. As shown in Figure 1B, the mice in OVA group exhibited the highest scratching frequency after injection. There was no significant difference in the scratching frequency of mice between OVA and BP groups. However, the mice in 2E-BP and 3E-BP groups exhibited significantly less scratching frequency than the mice in OVA and BP groups. This indicated that enzyme-treated bee pollen alleviates mice scratching behavior, and that enzymatic treatment can reduce the allergenicity of bee pollen.

### 3.2. Enzyme-Treated Bee Pollen Mitigates Histopathological Injury in Mice

**Figure 2:**
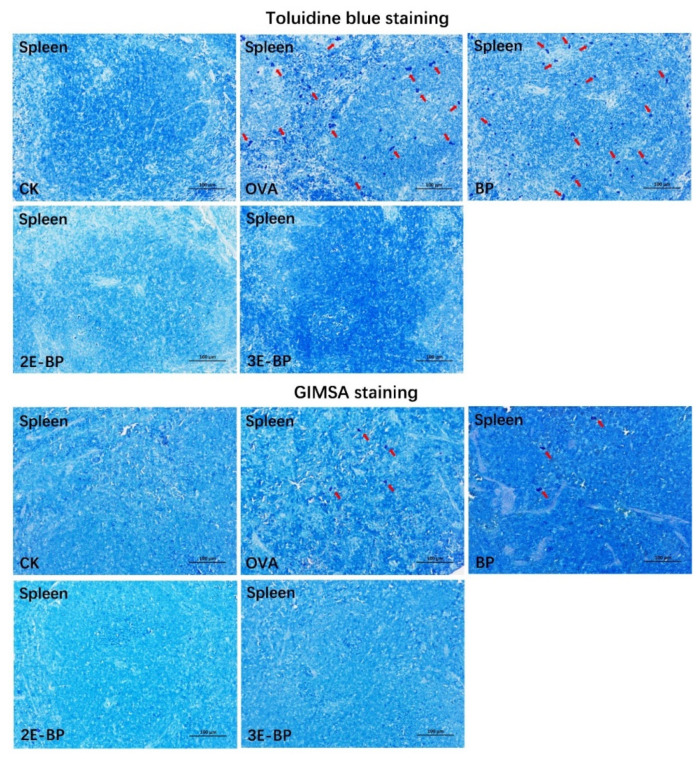
Histopathological changes in mouse spleen in different treatment groups. Mast cells and granulocytes stained separately with toluidine blue (TB) and GIMSA are marked with red arrows.

Mast cells and granulocytes play important roles in food allergy. Specifically, FcεRI as a kind of IgE receptor existing in mast cells and basophils can be activated by crosslinking with allergen-specific IgE antibodies, leading to the release of allergic mediators responsible for the early- and late-phase of allergic reactions [16,34,35]. Herein, the mast cells and granulocytes in mice spleen were visualized by TB and GIEMSA staining, respectively. As shown in Figure 2, there are significantly more mast cells and granulocytes (marked with red arrows) in OVA and BP groups than in CK group, while there is no significant difference in the 2E-BP and 3E-BP groups compared with the CK group. This revealed that enzyme-treated bee pollen mitigates histopathological injury in mice.

### 3.3. Enzyme-Treated Bee Pollen Decreases the Production of IgE Antibodies in Mice Serum

**Figure 3:**
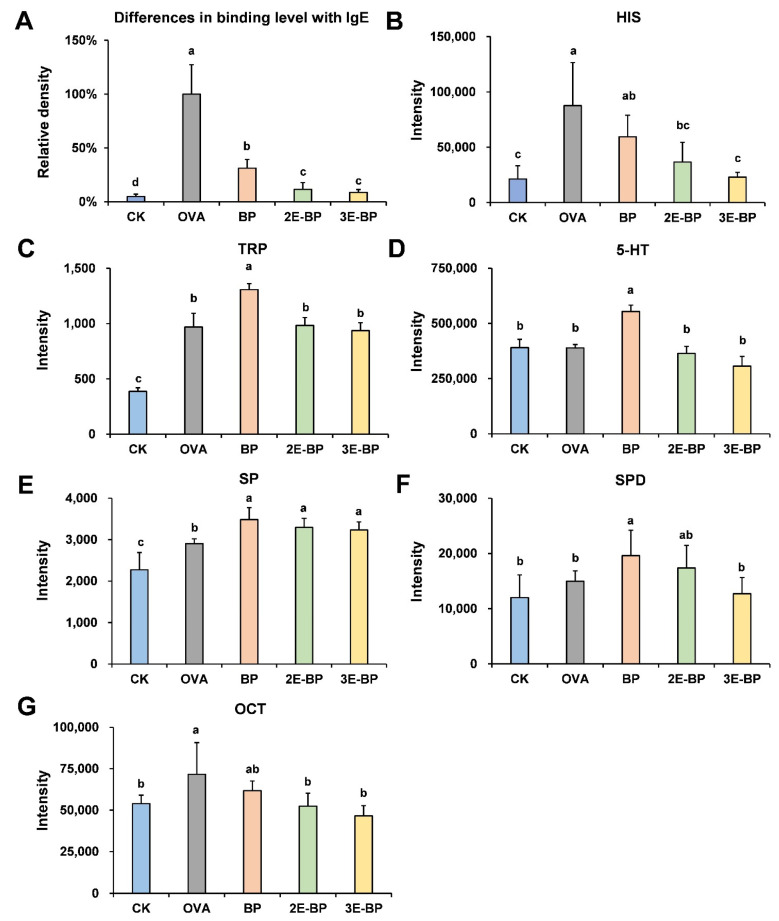
Changes in allergic mediators including IgE antibody (**A**), histamine (**B**), tryptamine (**C**), serotonin (**D**), spermine (**E**), spermidine (**F**) and octopamine (**G**) in mice serum of different treatment groups. Different letters indicate a significant difference among different groups (*p* < 0.05).

Generally, food allergy is an IgE-mediated type I hypersensitivity, which can induce immune cells to produce IgE antibodies [36,37,38]. Dot-blot assay was used to semi-quantitatively analyze the level of IgE antibodies in mice serum. As shown in Figure 3A, OVA and BP treatment induced a significant increase in mice serum IgE levels. However, mice serum IgE levels in the 2E-BP and 3E-BP groups exhibited a notable decrease compared with that in BP group. This suggested that enzyme-treated bee pollen could decrease the production of IgE antibodies in mice serum due to a reduction in allergic reactions.

### 3.4. Enzyme-Treated Bee Pollen Modulates the Bioamine Level in Mice Serum

Serum bioamines are critical indicators that reveal the allergic status of the body. The UPLC-QQQ-MS/MS technique was conducted to discover the changes in levels of bioamines in mice serum. Histamine (HIS) is a key allergic mediator released by mast cells and basophils, which can regulate T helper (Th) lymphocytes to produce inflammatory cytokines [39,40]. As shown in Figure 3B, the HIS level in the OVA and BP groups was significantly higher than that in the CK group; while there was no significant difference among the CK, 2E-BP and 3E-BP groups. Regulating T lymphocytes (Treg) contributes to the acquisition of allergy tolerance [41]. Tryptamine (TRP) and 5-Hydroxymethyltryptamin (5-HT) can interact with immune cells, triggering the conversion of Tregs to Th17 cells [42]. As shown in Figure 3C,D, both the levels of TRP and 5-HT were significantly higher in the BP group than that in the CK, 2E-BP and 3E-BP groups, suggesting that 2E-BP and 3E-BP could alleviate allergic reactions in mice. Spermine (SP) and spermidine (SPD) have been reported to provide protective effects by inhibiting the development of allergic asthma [43]. As shown in Figure 3E,F, the level of SP in BP, 2E-BP and 3E-BP groups was significantly higher than that in the CK and OVA groups; and the level of SPD in BP and 2E-BP groups was significantly higher than that in the CK, OVA and 3E-BP groups. This was attributed to the fact that bee pollen contains a certain amount of SP and SPD which can increase their levels in serum after ingestion [44]. Octopamine (OCT) is also one of the allergic mediators with proinflammatory effects to the body [45]. As shown in Figure 3G, the OVA and BP groups exhibited a higher level of OCT than the CK, 2E-BP and 3E-BP groups, indicating the alleviatory effect of 2E-BP and 3E-BP on food allergy.

### 3.5. Enzyme-Treated Bee Pollen Regulates Metabolism in Mice Serum

**Figure 4:**
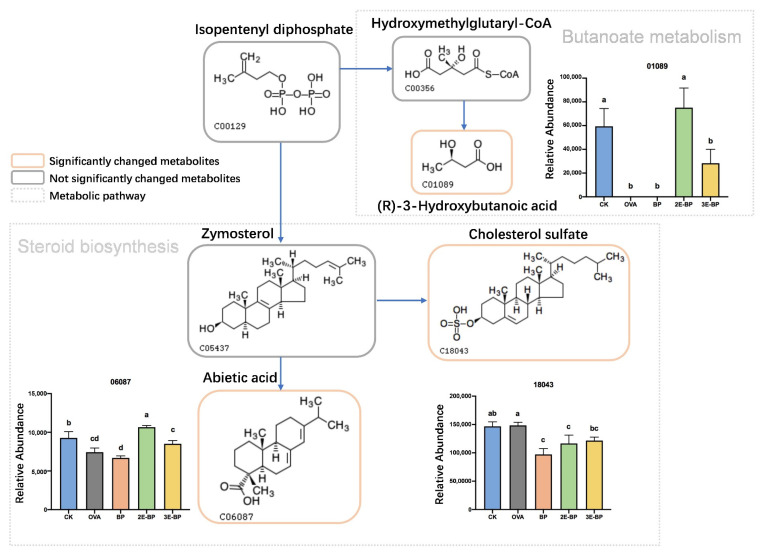
The changed metabolites and involved metabolic pathways in mice serum of different treatments groups. Different letters indicate a significant difference among different groups (*p* < 0.05).

Food allergy can induce metabolic disorders. Herein, the metabolomics analysis of mice serum was performed to explore the metabolism changes in mice serum. The metabolites with significant changes (*p* < 0.05; Fold Change > 2) among different groups were screened and enriched into corresponding pathways. As shown in Figure 4, the contents of (R)-3-hydroxybutanoic acid and abietic acid in CK, 2E-BP and 3E-BP groups were significantly higher than that in the OVA and BP groups; while the content of cholesterol sulfate in BP, 2E-BP and 3E-BP groups was notably lower than that in the CK and OVA groups. All of these significantly changed metabolites were enriched in two main metabolic pathways: (1) Steroid biosynthesis; (2) Butanoate metabolism.

As reported, steroid hormones exert various immunologic functions, for instance, steroid hormones can alleviate the clinical symptoms of allergic asthma [46,47]. Additionally, steroid hormones can contribute to the production of specific T cells, thereby exhibiting anti-inflammatory effects [48]. Butanoate metabolism was also closely associated with immune system function, for instance, butyrate helps enterocytes maintain their functionality and the integrity of intestinal mucosa, thereby preventing inflammation caused by pathogens [49]. Moreover, butyrate can reduce enterocyte inflammation by defending against oxidative stresses [50]. Moreover, the reduction of butyrate caused by the imbalance and dysfunction of gut microbiota leads to the aggravation of allergic reactions [51]. Therefore, enzyme-treated bee pollen might reduce the allergic reactions by regulating steroid biosynthesis and butanoate metabolism in mice serum.

### 3.6. Enzyme-Treated Bee Pollen Regulates the Composition of Gut Microbial Structures

**Figure 5:**
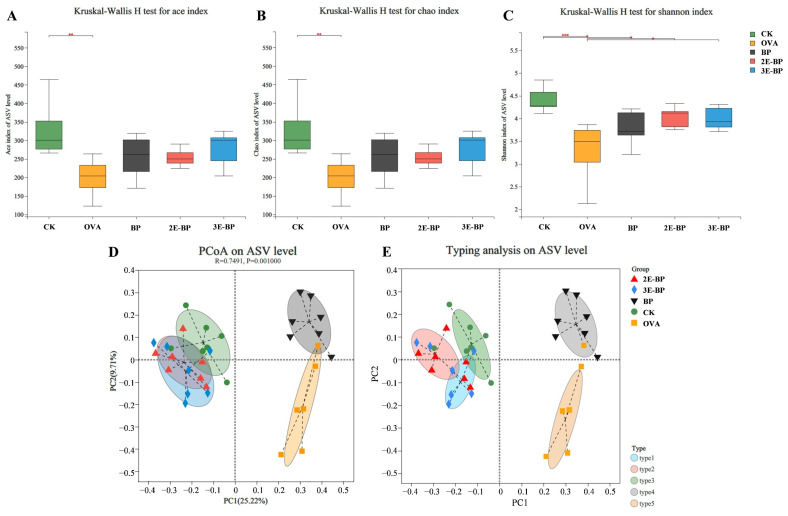
The alpha-diversity and beta-diversity analysis of different samples at ASV level, including (**A**) Ace index analysis; (**B**) Chao index analysis; (**C**) Shannon index analysis; (**D**) PCoA analysis; (**E**) Typing analysis. Asterisk * represents *p* < 0.05, ** represents *p* < 0.01, and *** depicts *p* < 0.001.

**Figure 6:**
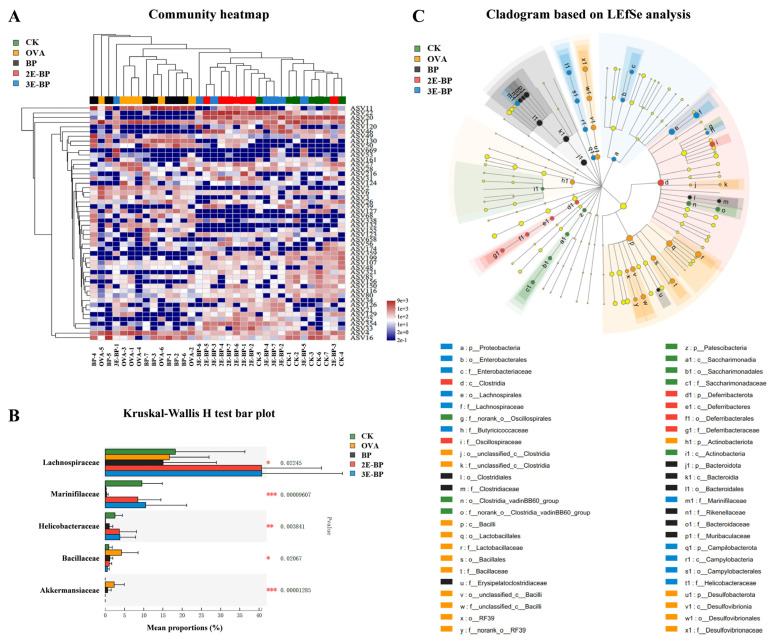
Heatmap analysis of gut microbiota community at ASV level (**A**). Kruskal–Wallis H test analysis of gut microbiota at family level (**B**). Cladogram based on LEfSe analysis of gut microbiota from phylum to class level (**C**). Asterisk * represents *p* < 0.05, ** represents *p* < 0.01, and *** depicts *p* < 0.001.

Furthermore, food allergy can induce an imbalance of gut microbiota associated with the immune system. As shown in Figure 5A,C, the Ace, Chao and Shannon indices were higher in the BP, 2E-BP and 3E-BP groups than that in the OVA groups, indicating that bee pollen can increase α-diversity of gut microbiota in mice. As shown in Figure 5D,E, 2E-BP and 3E-BP groups were well-separated with OVA and BP groups, suggesting the significant changes in β-diversity of gut microbiota. Additionally, clustering analysis showed that the OVA and BP groups had similar microbial structures, while the CK, 2E-BP and 3E-BP groups exhibited analogous microbial structures (Figure 6A). In consideration of the above findings, a severe allergy was induced by OVA and BP but was alleviated by 2E-BP and 3E-BP, thereby leading to a similar microbial structure among CK, 2E-BP and 3E-BP groups. Kruskal–Wallis H test and LEfSe analysis showed that *Lachnospiraceae*, *Marinifilaceae* and *Helicobacteraceae* were significantly more abundant in the CK, 2E-BP and 3E-BP groups than in the OVA and BP groups; while, the abundance of *Bacillaceae* and *Akkermansiaceae* was significantly lower in the CK, 2E-BP and 3E-BP groups than in the OVA and BP groups (Figure 6B,C).

As reported, *Lachnospiracea* could be involved in food allergies [52]. Its abundance was significantly increased after the allergic mice received an allergen-specific Treg cell therapy compared with the no-treatment group [53]. *Lachnospiraceae* level was also significantly reduced in the gut microbiota of allergy sufferers compared with healthy population [54]. *Marinifilaceae* can be affected by intenstinal inflammation. Its abundance was reduced in the gut microbiota of colitis mice but recovered following anti-inflammatory therapy [55]. *Helicobacteraceae* shows beneficial effects against food allergy, for instance, the neutrophil-activating protein of *Helicobacter pylori* can inhibit peanut allergy by up-regulating the production of Tregs [56]. The level of *Bacillaceae* is related to gut inflammatory diseases [57]. It presented a higher level in the gut microbiota of Crohn’s disease patients compared with healthy people [58]. Additionally, the level of *Akkermansiaceae* increased due to inflammatory gut injury and other gastorintestinal diseases [59]. Therefore, our findings correspond with those of previous reports. Enzyme-treated bee pollen can alleviate allergic reactions and regulate the composition of microbial gut structures.

## 4. Conclusions

Overall, in comparison with natural bee pollen, enzyme-treated bee pollen can reduce mice scratching frequency, spleen pathological injury, and serum IgE production. Moreover, it can additionally regulate bioamine serum levels, as well as modulate steroid biosynthesis and butanoate metabolism. Further, enzyme-treated bee pollen can regulate the composition of gut microbial structures by increasing the abundance of *Lachnospiraceae*, *Marinifilaceae* and *Helicobacteraceae*, while decreasing the abundance of *Bacillaceae* and *Akkermansiaceae*, which are involved in allergenicity mitigation. The findings suggest that enzymatic treatment has an alleviatory effect on the allergenicity of bee pollen, and provide a scientific basis for further development and utilization of hypoallergenic bee pollen products.

## Data Availability

Data available on request due to privacy.

## Supplementary Materials

The following supporting information can be downloaded at: https://www.mdpi.com/article/10.3390/foods11213454/s1, Table S1. The parameters for bioamines detection by UPLC-QQQ-MS/MS.
