# Supplementary material for: Allergenicity Alleviation of Bee Pollen by Enzymatic Hydrolysis: Regulation in Mice Allergic Mediators, Metabolism, and Gut Microbiota
Source: Foods. 2022 Oct 31;11(21):3454. doi: 10.3390/foods11213454 (PMC9658975; doi:10.3390/foods11213454)
Supplement: Supplementary file 1 [file foods-11-03454-s001.zip › foods-1968649-supplementary.pdf]

Supplementary

Table S1. The parameters for bioamines detection by UPLC-QQQ-MS/MS.

Compound	Precursor ion	Product ion	Collision energy (V)	Fragmentor (V)
Histamine (HIS)	112.1	95.0*<br>68.0	15<br>25	80
Tryptamine (TRP)	161.1	144.0<br>117.0*	10<br>30	70
5-Hydroxymethyltryptamin (5-HT)	177.0	160.1*<br>132.1	10<br>25	70
Spermine (SP)	203.2	129.0<br>112.0*	10<br>20	100
Spermidine (SPD)	146.2	129.0<br>72.0*	8<br>15	80
Octopamine (OCT)	154.1	136.0<br>91.0*	4<br>22	60

*means this product ion was used for quantification.
